# Model-Based Fault Diagnosis and Fault Tolerant Control for Safety-Critical Chemical Reactors: A Case Study of an Exothermic Continuous Stirred-Tank Reactor

**DOI:** 10.1021/acs.iecr.3c01205

**Published:** 2023-08-21

**Authors:** Pu Du, Joshiba Ariamuthu Venkidasalapathy, Sunjeev Venkateswaran, Benjamin Wilhite, Costas Kravaris

**Affiliations:** † Artie McFerrin Department of Chemical Engineering, Texas A&M University, College Station, Texas 77843, United States; ‡ Viking Engineering LC, Houston, Texas 77079, United States

## Abstract

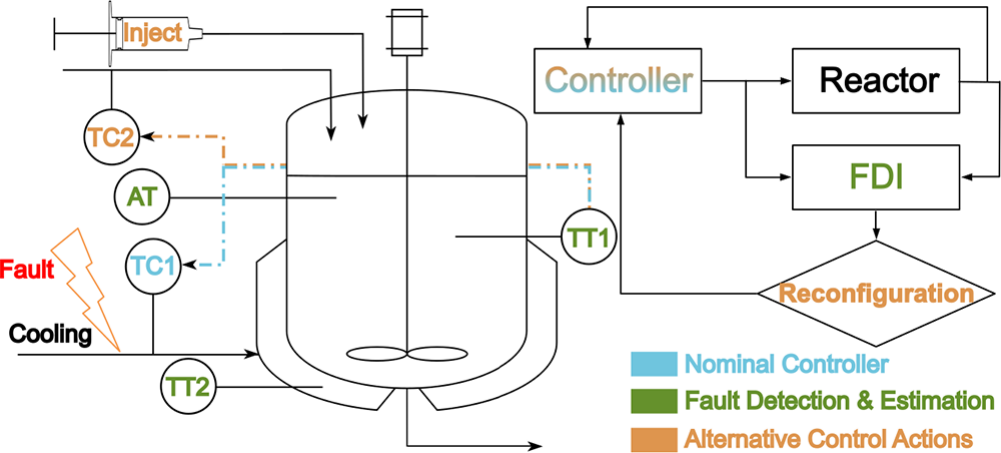

In safety-critical chemical reactors with potential hazards, reaction kinetics and heat transfer parameters are usually known, and a mathematical model is available. It is then meaningful to base fault detection and isolation algorithms on the first-principles model as opposed to statistics, so that physically meaningful residual signals are generated from material and/or energy balances not closing, leading to reliable fault diagnosis. Additionally, to maintain the safety of the entire system, it is necessary to take appropriate control action based on the mathematical model and the identified faults, to minimize their impact and thus ensure safe operation. In the present work, these ideas will be formulated and illustrated through a continuous stirred-tank reactor case study involving the liquid-phase oxidation of alkylpyridine with hydrogen peroxide. The proposed fault tolerant control strategy monitors the DSM (distance of the system state from the boundary of the dynamic safe set) and the estimate of the fault size, and when they cross a certain limit as a result of an abnormal event, the manipulated input is switched. Simulation results show the effectiveness of the proposed fault tolerant control strategy in dealing with cooling system failure.

## 1 Introduction

The increasing complexity of chemical processes poses a safety challenge, which in turn has motivated the development of fault tolerant control (FTC). The utilization of FTC methods in systems is expected to enhance process safety and reliability while minimizing the adverse economic consequences associated with failures in overall process operation.^1^ Generally, FTC strategies can be categorized into passive or active. These two alternative approaches emerge from different design philosophies. In the blueprint, a system is built fail-safe against certain predefined fault sets with built-in redundancy, rather than using real-time data from fault detection and isolation (FDI), and this is called passive FTC.^2−4^ Passive FTC suffers from conservative performance as it has a duty to handle both normal operation and faulty situations.^5,6^ On the other hand, active FTC is composed of the following ingredients: FDI, a reconfiguration scheme and an adaptable controller. FDI is of paramount importance in active FTC, and the adaptive reconfiguration of the controller to new operating conditions is based upon the detection and isolation of faulty components with FDI, so active FTC could be tuned with optimal performance for each fault scenario, depending on the outputs of FDI; this is a major advantage of active FTC.^7,8^

In the past 30 years, there have been many endeavors in active FTC. The linearization of a system close to the operation conditions is the widely applied technique to synthesize controllers, a traditional approach.^9,10^ Besides linearization, strategies to reconfigure a controller include back-stepping,^11^ feedback linearization,^12^ dynamic inversion,^13^ Lyapunov methods,^14^ and most recently, neural networks.^15,16^ Yet all these methods meet major drawbacks in constrained states and inputs. To tackle such a problem, a typical practice to rebuild a controller is based on the stability region derived from a level set of Lyapunov functions for the fall-back configuration.^17,18^ However, the level sets of Lyapunov functions may form an overly conservative estimate of the stability region, limiting the backup reorganization algorithm of controllers. In addition, if we had the FDI schemes and fault estimators, we could prevent unnecessary switching of controller configurations by utilizing such information.

In a chemical process system, a fault is a deviation of one or more parameters from the nominal value beyond the acceptable threshold. If a fault is not properly contained, it might turn into a failure or incapacitate the system. Thus, the main purposes of FDI include (i) the detection of malfunctions synchronously, (2) isolating the faulty components, and (3) estimating the nature of the fault.^19^ This practice can be classified as either hardware redundancy or analytical redundancy.^20,21^ Despite its reliability, the cost of hardware redundancy is high, and it is not practical to build an identical system only for fault diagnosis, not to mention that in a tailored system, replication of identical hardware components is challenging from the beginning. On the other hand, analytical redundancy compares the actual system’s behavior to a nominal system model, the expected behavior, and records any deviations above a certain threshold, through the so-called residual generators.^22^ In recent times, there has been a notable increase in studies focusing on the detection and isolation of actuators and sensor faults using residual generators.^23,24^

For most safety-critical chemical reactors, the dynamics of the system is known. The question is whether the concealed disturbances and hidden uncertainties,^25^ e.g., reaction rate fluctuations, invalidate the residual signals. There are several models in FDI, including Kalman filters,^26^ parity equations,^27^ parameter estimation,^28^ structural analysis,^29^ and observers, out of which the observer-based fault diagnosis schemes are the most prevailing. Pioneered by Beard and Jones in the 1970s,^30,31^ observer-based FDI approaches have been flourishing, mostly based on Luenberger observers^32^ and Kalman filters. However, it was not until the early 21st century, that observer-based FDI can handle nonlinear systems, led by the work of De Persis and Isidori.^25^ In chemical reactions, the kinetics has an exponential term depending on temperature; thus, nonlinearities are inherent in chemical process models. A powerful methodology for building observers for nonlinear systems is based on exact observer error linearization,^33^ and this point of view has been recently followed to build observer-based FDI for nonlinear systems, where for each fault, the residual signal is independent of other faults and disturbances.^34^

As mentioned earlier, Lyapunov functions generally give rise to conservative estimates of stability regions, limiting the capacity for adjustment of controller parameters. From another perspective, the overall safety region for an entire process could be characterized by a set of constraints that should be satisfied at all times to prevent apparent faults or failure. A process that violates such constraints is marked as undesirable and fault correction should be applied immediately, for example, the temperature of a reactor excessing the preset limit, the pressure of a pipe is over the material property and the load of a column might reach saturation. Besides these limits of unit operations, the available control inputs are vital for system safety, as the system’s robustness and resilience rely on these inputs. The problem is how to integrate these safety regions with the parameter space of controllers. There have been several studies aiming at a comprehensive integration of safe operation and controller designs,^35^ most of which define safety as an additional set of constraints in a model predictive control (MPC) framework by solving dynamic optimization in real-time. Lyapunov-MPC has been presented recently,^36^ and advanced MPC designs have demonstrated their effectiveness in mitigating the growing cybersecurity threats in the chemical industries.^37^ The concepts of a dynamic safe set (DSS), defined as a maximal admissible set, and dynamic safety margin (DSM), defined as the distance from the boundary of the DSS, play a critical role in formulating and designing the control strategy.^38^ Monitoring the position of the system in the interior of the DSS, including the size of the DSM, provides criteria for adapting control action after the occurrence of an abnormal event.^39^ A systematic study of T-2 reaction design with maximally safe control was based on DSM.^40^ In addition, it is possible to further investigate fault tolerant control with an accurate yet nonconservative estimation of the safe region of each controller in the presence of a fault, combined with on-the-fly FDI.

Alkylpyridine N-oxidation with hydrogen peroxide as the oxidizer is an important synthesis step for pharmaceutical intermediates and other useful organic chemicals. This reaction is highly exothermic with the possible decomposition of hydrogen peroxide generating oxygen, raising safety concerns.^41^ Inherently safer operation conditions were studied based on the boundary diagrams.^42^ Recent research has shown that continuous operation in a continuous stirred-tank reactor (CSTR) is advantageous in controllable temperature profiles and concentration homogeneity, suitable for this specific reaction.^43^

In this study, we are proposing a new FTC strategy where DSS monitoring is integrated into the FTC design. This incorporation allows us to obtain nonconservative estimates of safety regions, which cannot be achieved using traditional Lyapunov methods. Additionally, our proposed FDI technique is based on error linearization leading to robustness against uncertainties in reaction rates that are not explicitly bounded. This type of uncertainty is particularly prevalent in chemical reactions and presents major challenges. By incorporating these advancements, we obtain significant improvements in the operation and performance of reaction systems, enhanced process safety, and reliability, as well as a reduction in the negative economic impact caused by failures in overall process operation. Specifically, this work proposes an active FTC scheme for an exothermic CSTR, driven by FDI and performing switchings between alternative control configurations. We start with a study of diagnosability that defines two groups of faults during the operation of the CSTR. We have found that one fault from each group can be effectively isolated via appropriate residual generators, despite persistent disturbances in the reaction rate, both in the open loop and in the closed loop under temperature control that manipulates the coolant flow rate or coolant inlet temperature. Next, focusing our attention on a coolant flow rate fault as it is most detrimental to system safety, we devised two alternative strategies to tackle it, switching the controller to reduce reactant feed and emergent solvent injection to dilute the reactants in the CSTR. During the operation of the reactor, state estimates from a nonlinear observer track the system in state space and determine the corresponding DSS. With the fault estimator tracking the fault size and calculating DSM, we applied alternative fault handling strategies; under a smaller fault estimate size the manipulated input was switched to feed flow rate and for a larger one we deployed solvent injection. By checking the system trajectories in terms of DSM and fault-size estimates, we derived switching criteria for the FTC scheme.

## 2 Background

This section will provide a brief necessary review of two key ingredients of the proposed fault tolerant control strategy: (a) the dynamic safe set (DSS), within which the system trajectory must lie at all times, in order to satisfy the safety constraints and (b) the residual generator and fault estimator to be used for detecting, isolating, and estimating the size of the fault, in order to assess its potential impact and decide on appropriate corrective action.

### 2.1 Dynamic Safe Set and Dynamic Safety Margin

Consider a discrete-time linear system with state *x*(*t*) and output *y*(*t*) of the form eq 2.1.1, which is subject to a disturbance input *w*(*t*) that lies within a bounded set **W** at all times
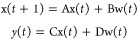
2.1.1and suppose that the output must lie in a set **Y**, in order to satisfy safety constraints. Then the maximal admissible set^44,45^
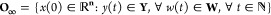
2.1.2is the set of all initial conditions *x*(0) that result in the output *y*(*t*) lying within the constraint set **Y** at all times, for all disturbance inputs *w*(*t*) in **W**. The set **O_∞_** given by eq 2.1.2 is called the DSS and plays a key role in designing controllers from a safety perspective.^40^

To quantify the level of safety at a given point in time, one can calculate the dynamic safety margin (DSM).^40^ DSM is defined as the distance from the boundary of the DSS, and its value is an indicator of how far is the system from violating the safety constraints. Computational algorithms are available for calculating **O_∞_**, from which the DSM can be readily calculated via the projection theorem.^40^

### 2.2 Linear Residual Generator and Fault Estimator

A systematic method for designing linear residual generators of the form
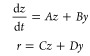
2.2.1has been recently developed^34^ for detecting and isolating faults in nonlinear systems of the form
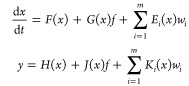
2.2.2where  stands for the state vector,  are the output measurements,  is a fault acing on the system and  are disturbances. The residual generator generates a signal *r*(*t*), which remains at zero in the absence of faults, becomes nonzero in the presence of a fault and is unaffected by the disturbances *w_i_*. In particular, it has the following properties:

- *r*(*t*) approaches zero asymptotically, under any initial conditions, when *f* is zero
- *r*(*t*) is disturbance-decoupled, i.e., unaffected by *w_i_*
- *r*(*t*) is sensitive to *f*.

Such a residual generator can be constructed under the following conditions,^34^

(i) There exist constant row vectors  (called parity vectors) that satisfy:

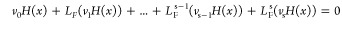
2.2.3where *L*_F_ is the Lie derivative operator, .
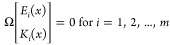
2.2.4
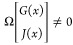
2.2.5where Ω is given by
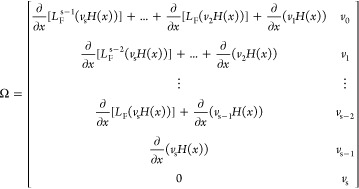


In the presence of multiple faults, one residual generator will have to be built for each fault, unaffected by the process disturbances and the other faults (these are all viewed as disturbances). In this way, faults can be isolated.

Furthermore, it is possible to construct a functional observer to estimate the size of a fault if the nature of the fault (*i*step, ramp, etc.) is known. In particular, defining an exo-system that generates the type of fault under consideration and connecting it with the original system (eq 2.2.1) gives rise to an extended system
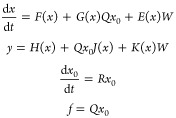
2.2.6for which a linear functional observer can be built under appropriate (similar to eqs 2.2.3–2.2.5) conditions.^46,47^

Common types of faults include (i) bias, an offset in the actual magnitude from the nominal value, usually a constant over time; (ii) drift, a gradual shift over time; (iii) loss of accuracy, involving a random degradation from the design value; and (iv) freezing, a nonzero constant independent of time.^48^

For example, a bias fault could be represented as a step function (*R* = 0, *Q* = 1 in eq 2.2.6) and a drift fault as a ramp ( in eq 2.2.6), leading to a corresponding fault estimation algorithm based on a disturbance-decoupled functional observer.

## 3 CSTR Model

**Figure 1:**
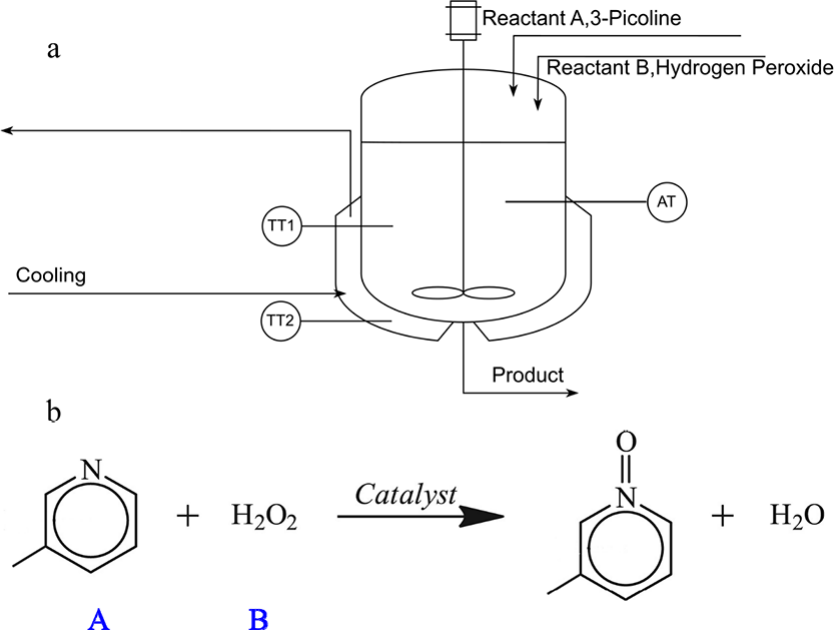
(a) CSTR reactor and (b) 3-picoline N-oxidation reaction.

Consider a CSTR system as depicted in Figure 1a, involving two feed flows for two reactants, 3-picoline, marked as A, and hydrogen peroxide, marked as B, respectively, and one coolant flow to cool down the highly exothermic reaction. The chemical equation is depicted in Figure 1b. In this N-oxidation reaction, it is generally preferred to have the oxidizer in excess, so the molar feeding ratio is λ = 1:1.05 (3-picoline versus hydrogen peroxide).

### 3.1 Open-Loop CSTR Model

The open loop CSTR model consists of mass balances and energy balances^43^ and is given by eqs 3.1.1–3.1.4 below, in the absence of faults or disturbances. There are three sensors to monitor the reaction, so the system outputs are given by eqs 3.1.5–3.1.7.
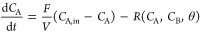
3.1.1
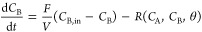
3.1.2
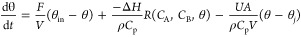
3.1.3
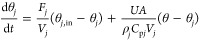
3.1.4
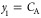
3.1.5
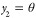
3.1.6
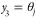
3.1.7

In eq 3.1.1–3.1.3, the reaction rate is denoted as *R*(*C*_A_, *C*_B_, θ), and the specific reaction rate expression is given in eq 3.1.8, based on kinetic studies from the literature, with kinetic parameter values given in Table S1 in the Supporting Information.^41,49^
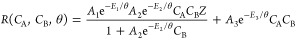
3.1.8

**Figure 2:**
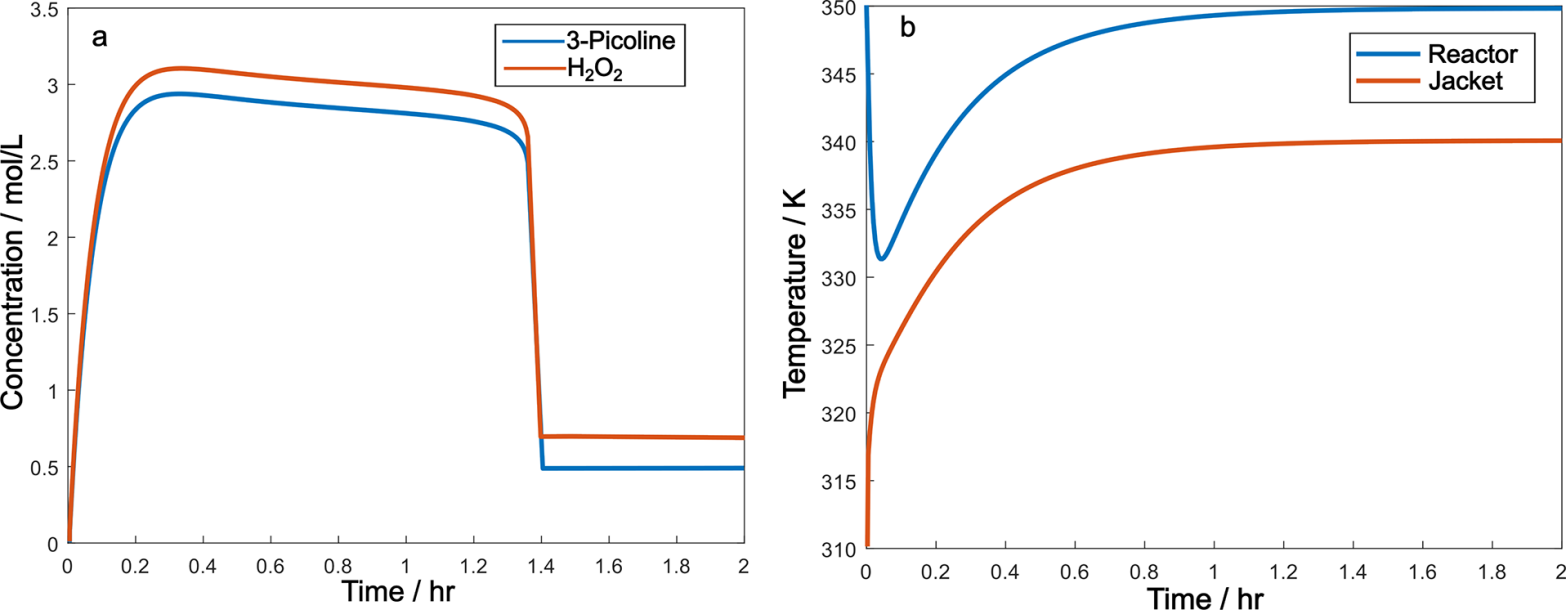
Open-loop concentrations vs time (a) and temperatures vs time (b).

The simulation design parameters for the CSTR system including the physical properties are provided in Table S2 in the Supporting Information. These parameters are carefully chosen to ensure efficient reaction conversion while also maintaining sensitivity to faults. Based on these design parameters, which correspond to reasonable operating conditions, we have simulated the start-up of the CSTR, and the results are shown in Figure 2, indicating that within 0.2 h, the reactant concentrations are building up, and not much heat is generated, as we could see a decrease in reactor temperature due to heat transfer to the cooling jacket. After 0.2 h, the reaction prevails and the temperature of the reactor is steadily increasing until 1.4 h to reach a steady state; the reactant concentrations undergo an abrupt drop to their corresponding steady-state values. As seen in Figure 2, the temperature of the reactor is less than 375 K at all times; therefore, the risk of boiling and excess decomposition of hydrogen peroxide is avoided.^43^

### 3.2 Closed-Loop Response of Reactor Temperature under a PI Controller

**Figure 3:**
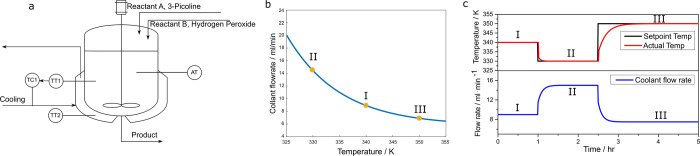
Reactor with a PI controller that regulates the reactor temperature by manipulating coolant flow rate (a), steady-state plot of reactor temperature vs coolant flow rate (b), and closed loop reactor temperature response under set point changes and corresponding coolant flow rate (c).

In industry, the temperature of a jacketed exothermic CSTR is usually regulated by manipulating the coolant flow, as shown in Figure 3a, and a PI controller is commonly used for this purpose. The steady-state plot of reactor temperature versus coolant flow rate for our CSTR system is shown in Figure 3b, from which it is clear that the reactor temperature is sufficiently sensitive to the coolant flow rate. We see that within a 14 mL/min flow rate interval, the steady-state reactor temperature could be operated within a 20 K span.

Under the action of a PI temperature controller, the jacket energy balance takes the form
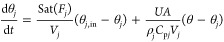
3.2.1where
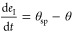
3.2.2
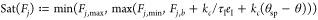
3.2.3

In order to tune this PI controller, the signature method^50^ was applied to the linearized closed loop equations, leading to a stabilizing set of controller parameters, within which the controller was tuned. The PI controller parameters are listed in Table 1.

**Table 1:** Parameters of the PI Controller Regulating Reactor Temperature

Parameter	Symbol	Value
controller gain	*k_c_*	–0.15
integral time	τ_I_	2
base flow rate	*F*_*j*,*b*_	7.5
upper limit	*F*_*j*,max_	10
lower limit	*F*_*j*,min_	0

To validate our PI controller tuning, we tested it through a series of step changes in the set point, as defined by eq 3.2.4 below. The system transitioned between three different steady states, with temperature variations up to 15 K, sufficiently large to challenge our controller.
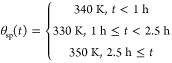
3.2.4

In Figure 3c, we see that for each change of temperature set point, the calculated coolant flow rate transitions smoothly without hitting the saturation limit, and the actual reactor temperature reaches the new set point around one residence time, which is fast, and without oscillation. This leads to the conclusion that the PI controller is both feasible and capable of managing the reactor temperature.

### 3.3 Possible Faults in the CSTR

**Figure 4:**
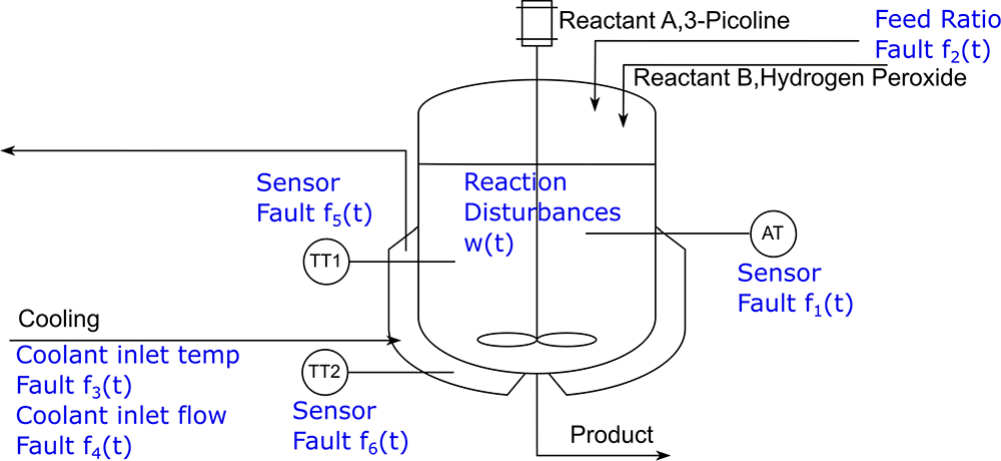
Possible fault points in our CSTR.

There is no process without possible faults, nor is the CSTR under consideration. The success of FDI relies on a comprehensive understanding of where the faults could be happening. In Figure 4, we marked all possible fault points in our CSTR, and they are summarized in Table 2.

**Table 2:** Summary of Possible Faults and Their Potential Consequences

Category	Location	Symbol	Consequence
sensor malfunction	AT	*f*_1_	inaccurate reading of 3-Picoline concentration (*C*_A_)
	TT1	*f*_5_	inaccurate temperature readings for θ and θ_*j*_
	TT2	*f*_6_	
reaction disturbance	reactor	*w*	reaction rate variation (*R*)
actuator flaw	feeding inlet	*f*_2_	molarity ratio changes (λ)
	cooling flow	*f*_4_	inadequate cooling flow (*F_j_*)
	coolant temperature	*f*_3_	insufficient cooling (θ_*j*,in_)

Table 2 indicates possible faults and their consequences. Considering that the most destructive incidents happening in a chemical plant usually involve unexpected cooling flow deficiency, as in the T2 Laboratories explosion,^51^ subsequent sections will have a special emphasis on the effect of cooling system faults.

## 4 Fault Detection, Isolation, and Estimation

### 4.1 Diagnosability

Table 2 indicated six possible different faults for the CSTR system and one reaction disturbance (uncertainty/variability in the reaction rate expression). From linear systems theory, it is known that the largest total number of isolable faults and disturbances is no more than the total number of sensors,^21^ and in our setup, there are three sensors, so in our system, in the presence of one disturbance, there are no more than two faults that are detectable and isolable. This raises the key question of diagnosability, which will be initially addressed from a purely physical perspective, and subsequently, it will be checked in terms of the feasibility and performance of the residual generators.

Consider now our process model, originally given by eqs 3.1.1–3.1.7, but now incorporating faults *f*_1_ – *f*_4_ in the model (for simplicity we did not include all six faults), along with the reaction rate disturbance w, as additional inputs:
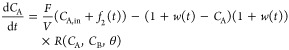
4.1.1
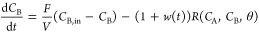
4.1.2
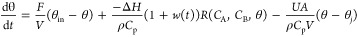
4.1.3
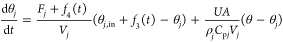
4.1.4
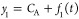
4.1.5
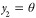
4.1.6
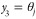
4.1.7where

- FTIR sensor fault, *f*_1_
- Feed ratio fault, *f*_2_
- Coolant inlet temp fault, *f*_3_
- Coolant inlet flow fault, *f*_4_
- Reaction disturbance, *w*.

The four faults can be categorized into two groups, as shown in Table 3, and we can detect and isolate one fault from each group, unaffected by the reaction disturbance, utilizing eqs 2.2.3–2.2.5.

**Table 3:** Fault Groups and Reaction Disturbance

Reaction disturbance	Fault group 1	Fault group 2
*w*(*t*)	sensor fault, *f*_1_	coolant inlet temp fault, *f*_3_
	feed ratio fault, *f*_2_	coolant flow rate fault, *f*_4_

Two faults from the same group violate the same balance, and this is the reason we cannot distinguish them. Group 1 faults, both affect component mass balance for reactant A, 3-picoline (see eqs 4.1.1 and 4.1.5). Group 2 faults enter in the same equation, eq 4.1.4, which represents the jacket energy balance – whether it is the coolant temperature or the cooling flow rate, the effect is decreased cooling capacity. Because these two physical variables have the same effect, they cannot be distinguished.

### 4.2 Residual Generators

The method of constructing linear residual generators outlined in Section 2.2 will now be applied to the CSTR system. For every pair of faults, one from each group, we can construct a residual generator for each fault, which is decoupled from the other fault and the disturbance w. If we were to select two faults from the same group, the conditions of Section 2.2 cannot be satisfied, and therefore, the faults cannot be isolated.

In what follows, as an illustration, we construct residual generators for *f*_1_(*t*): FT-IR detection fault and *f*_4_(*t*): Coolant flow fault, each one decoupled from the other fault and the disturbance *w*(*t*) of the reaction. For brevity, we wrote equations in deviation form, in which *C*_A_^′^ = *C*_A_ – *C*_A, s_, etc., the deviation from the corresponding steady-state value, which is given in Table 4.

**Table 4:** Steady-State Temperatures and Concentrations

Process variable steady-state	Symbol	Value	Unit
3-picoline concentration	*C*_A,s_	0.52	mol/L
hydrogen peroxide conc.	*C*_B,s_	0.68	mol/L
reactor temperature	θ_s_	350	K
jacket temperature	θ_j,s_	338	K

#### 4.2.1 Residual Generator 1: Coolant Flow Fault Detection

The following parity vectors satisfy the conditions of eqs 2.2.3–2.2.5 for *s* = 1:
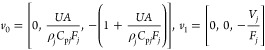
4.1.8

The design parameters are chosen as A = – 1, B = *v*_1_ – *v*_0_, C = 1, D = – *v*_1_, leading to
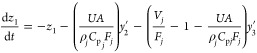
4.1.9
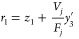
4.1.10

#### 4.2.2 Residual Generator 2: FT-IR Sensor Fault Detection

The following parity vectors satisfy the conditions of eqs 2.2.3–2.2.5 for *s* = 1:
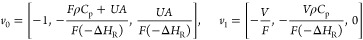
4.1.11

The design parameters are chosen as A = – 1, B = *v*_1_ – *v*_0_, C = 1, D = – *v*_1_, leading to
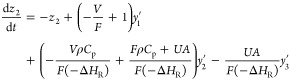
4.1.12
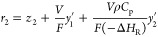
4.1.13

**Figure 5:**
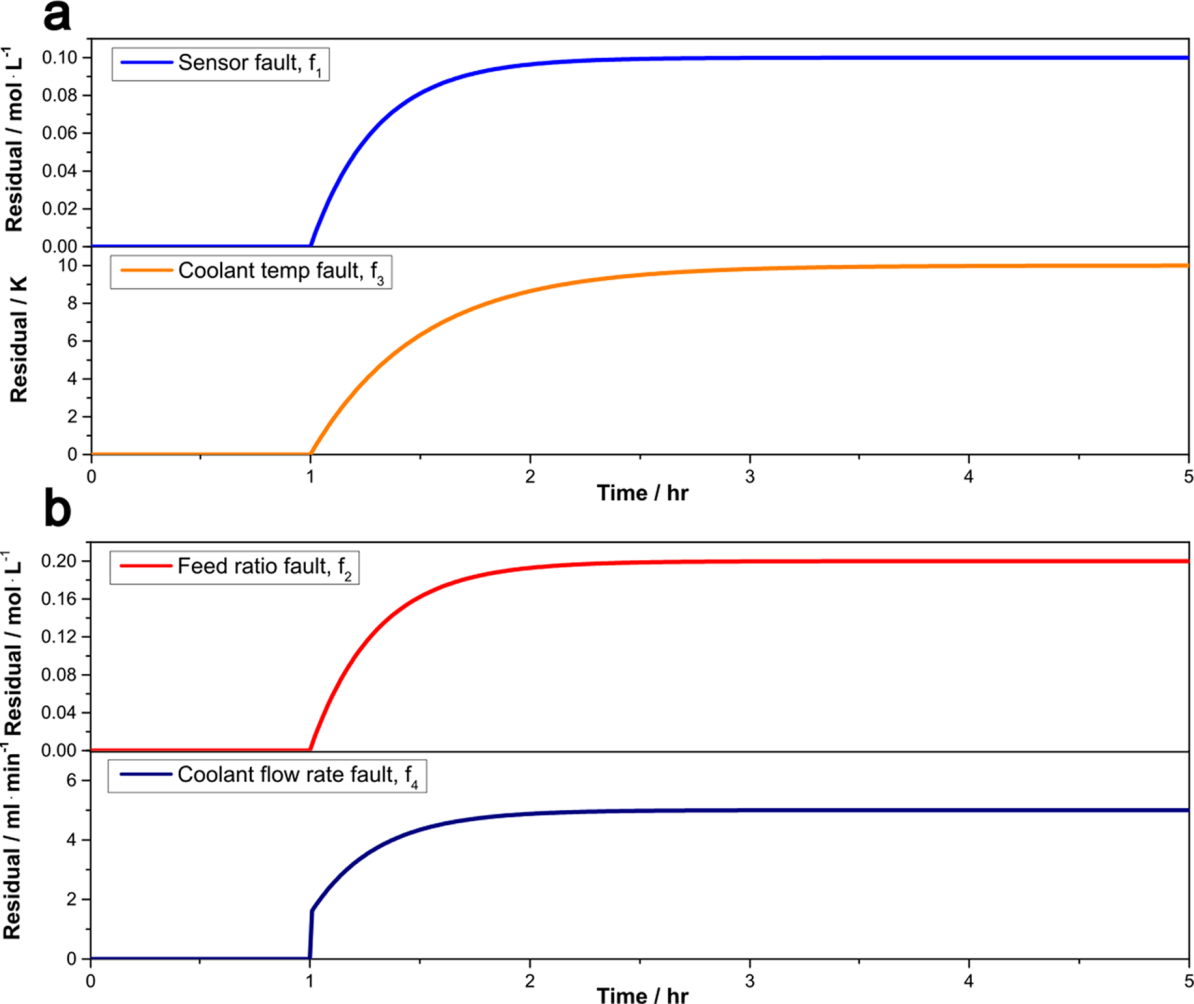
Response of residual generators for sensor fault and coolant inlet temperature fault (a), response of residual generators for feed ratio fault and coolant flow rate fault (b).

Similarly, we can construct residual generators for the other fault pairs, one from each group. The residual generator will give a zero signal in the absence of the fault that it is detecting and will approach the size of the fault asymptotically. Figure 5 provides test results for the residual generators for the following fault scenarios (*t* stands for time in hours).
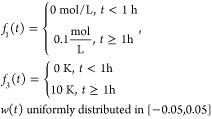
and,
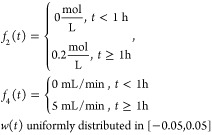


We see from Figure 5 that the residual signals remained zero before faults happened and started moving away from zero once the faults were introduced. After a long time, the residual signal approaches the magnitude of the fault. This suggests that our residual generators are not only indicators of faults happening, but they seem to act as estimators of the fault sizes; this will be discussed later in this section.

### 4.3 Residual Generators under Closed-Loop Conditions (PI Control of Reactor Temperature)

Under reactor temperature control, a coolant flowrate fault could induce control action saturation. In a closed loop, the energy balance on the jacket takes the form
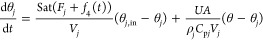
4.3.1where *F_j_* is the coolant flowrate calculated from the PI controller.

**Figure 6:**
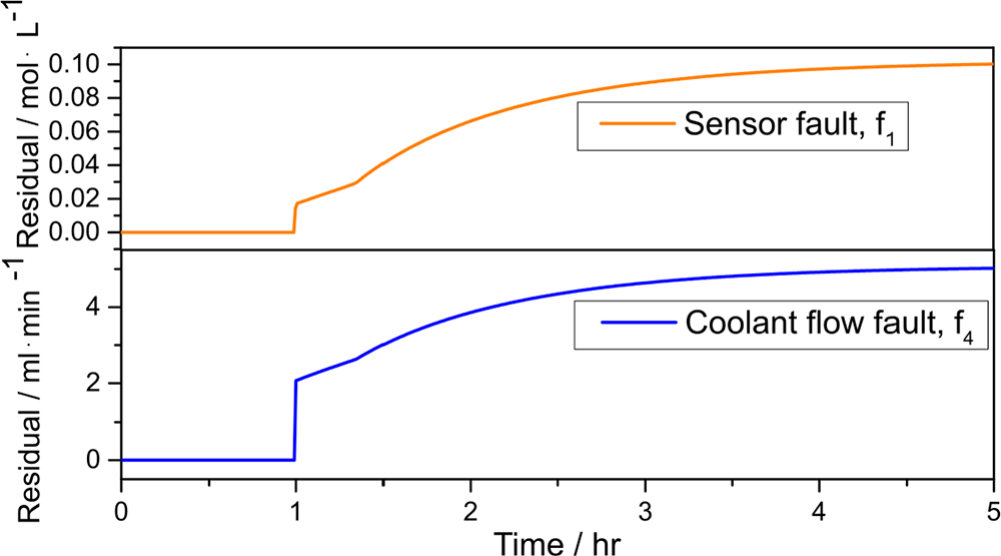
Residual generators with sensor fault and coolant flow rate fault in the closed loop.

**Figure 7:**
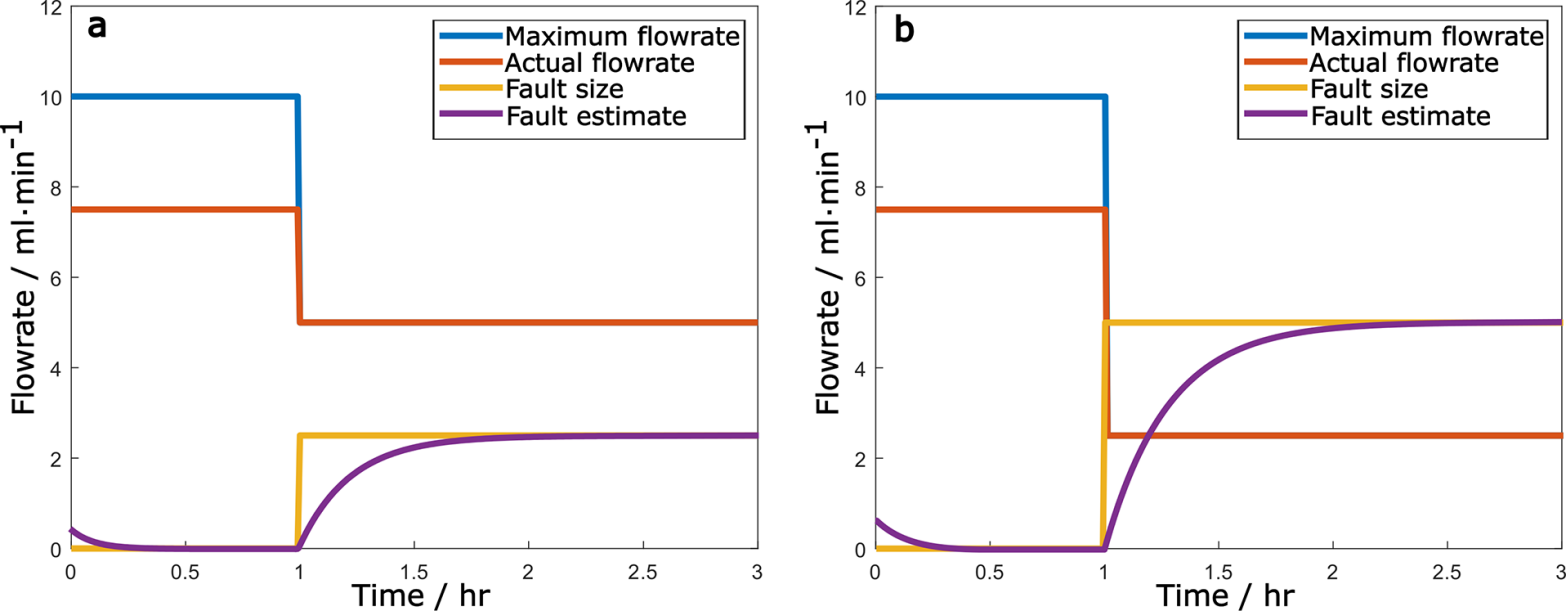
Coolant flow rate and fault estimation for 50% step reduction (a) and 75% step reduction (b) of pump capacity.

In what follows, we test residual generators under closed-loop conditions. The following fault scenario was simulated, and the result is shown in Figure 7:
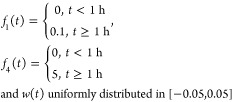


From Figure 6, we see that the residual generators performed well, both before and after the inception of faults, with the value of the residual approaching the magnitude of the faults asymptotically.

### 4.4 Fault Estimation

We constructed a fault estimator based on Section 2.2. Considering a drift fault (ramp) in coolant flow in eq 2.2.6, the following parity vectors satisfy the conditions for *s* = 1:
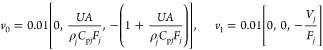
4.4.1and the functional observer with parameters *A* = – 0.01, *B* = 0.01*v*_1_ – *v*_0_, *C* = 1, *D* = – *v*_1_:
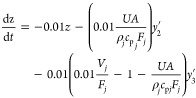
4.4.2
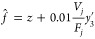
4.4.3

The above fault estimator for ramp fault is also applicable for the case of bias fault (step).

To test the fault estimator, we considered two faults on the maximum available coolant flow rate, a smaller one involving a 50% reduction and a larger one involving a 75% reduction in pump capacity. In the case of an abrupt change (bias fault), these could be defined as steps:
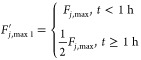
4.4.4
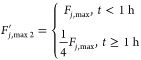
4.4.5where *F*_*j*, max_ = 10 mL/min is the maximum flow rate delivered by the pump under normal circumstances. The fault size is defined as coolant flow rate deviation from the design steady-state value of *F*_*j*, s_ = 7.5 mL/min, i.e., fault size = *F*_*j*, s_ – *F*_*j*, max_^′^.

From Figure 7a, it is seen that when the fault happens, the upper limit of the coolant flow rate is lowered, causing the actual flow rate to also be half of the upper limit. The estimator accurately estimates the fault size within 30 min. Similarly, from Figure 7b, the estimator is accurate as expected, and within 30 min, it gives the fault size estimate of 5 mL/min, exactly the deviation from the steady state.

In the same manner, two drift fault scenarios were considered in the coolant flow rate, as indicated in eqs 4.4.6 and 4.4.7, with units in ml/min. The first one involves the coolant flow rate drifting down to 50% capacity and the second down to 25% capacity.
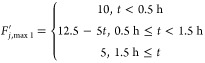
4.4.6
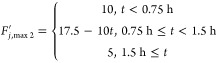
4.4.7

**Figure 8:**
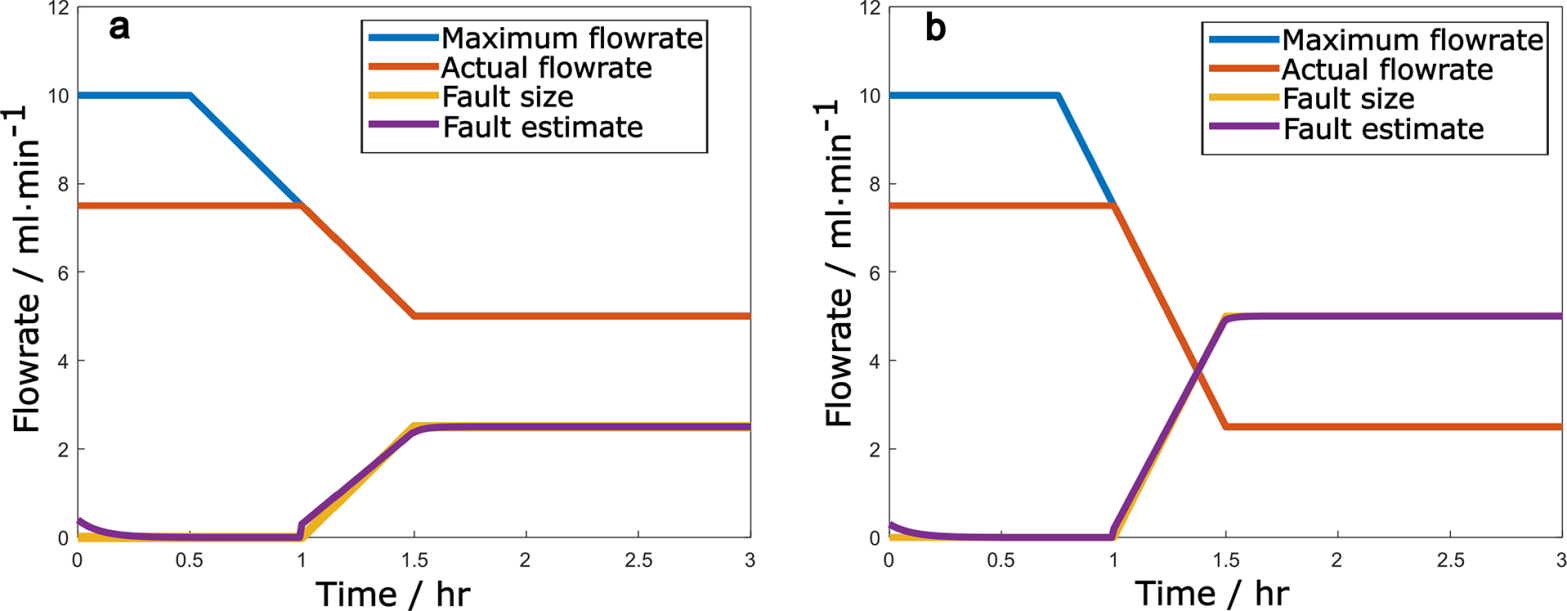
Fault estimation for coolant flow rate drifting down to 50% capacity (a) and down to 25% capacity (b).

From Figure 8, we see that the fault estimators perform remarkably well in terms of tracing the fault size, a swift response at the moment of the fault happening, and then climbing the slope of the drifting fault.

The simulation results of Figures 7 and 8 also indicate that the estimators perform the same way, irrespective of the size of the fault, which is because the estimators were designed to have linear dynamics. Additional simulations (not shown here) confirmed the consistent performance of the fault estimator for other fault sizes, including the case of 100% failure of the cooling line.

## 5 Fault Tolerant Control under Possible Coolant Flow Fault

In the previous section, we have demonstrated that there are means of detecting and estimating faults, which is critical to trigger appropriate action. In the present section, we will investigate how to design control action(s) that counteract a fault. First of all, we need to determine what kind of counteractions would be feasible and effective. In addition, we need to determine when, how, and under what conditions these actions will be implemented. Generally speaking, we need to have a “plan B” and possibly a “plan C” (in case plan B is not sufficiently effective) and specify the conditions under which they will be activated.

The present section will try to provide answers specifically for the case of a cooling system fault, as this represents the most threatening fault in the operation of an exothermic chemical reactor.

### 5.1 Possible Counteractions to Cooling System Fault: Testing Their Effectiveness

Once a fault happens, appropriate countermeasures must be implemented quickly, before adverse consequences arise. In order to specify these countermeasures, there must be a clear definition of the safety limits within which the process has to operate. Given the nature of the reaction under consideration, a reasonable safety specification is that the reactant should not exceed the boiling point, and it is known from previous studies that below 110 °C the decomposition of hydrogen peroxide is insignificant,^52^ so we set the safety constraint as reactant temperature below 375 K.

For an exothermic reaction, the most effective way to prevent overheating would be to add an inhibitor to quench the reaction, but unfortunately, to the best of our knowledge, there is no such compound that hinders the N-oxidation reaction without causing the decomposition of hydrogen peroxide, which could lead to an even worse catastrophe. Therefore, we examine two possibilities for counteracting a possible coolant flow failure:

(i) Reduction of feed flow rate, to reduce the reaction rate.
(ii) Injection of cold solvent, to dilute the reactor contents.

**Figure 9:**
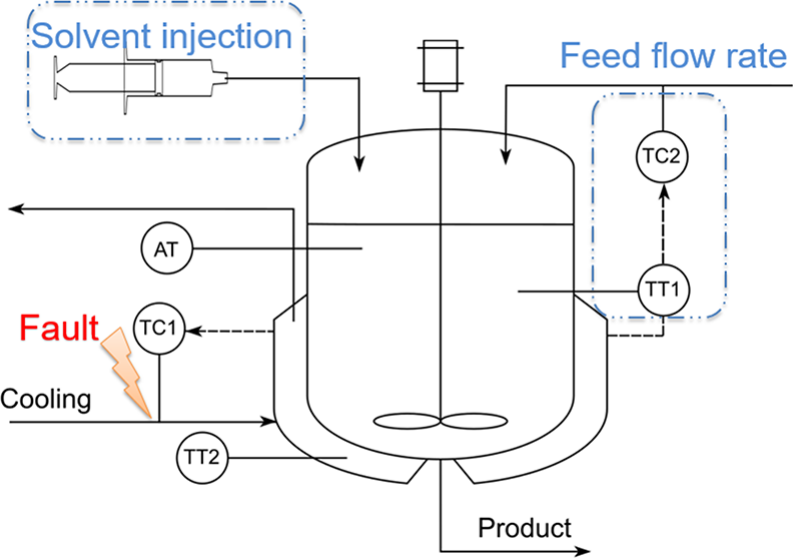
CSTR diagram indicating the cooling flow fault as well as possible counteractions.

The two possible counteractions under a coolant flow fault are illustrated in Figure 9, and the dotted areas indicate the control actions to be involved in the re-configured control system.

In order to evaluate the prospect of these counteractions in keeping the system within the safety limit, we have performed a simulation study, starting with the fault scenario of a 50% step reduction in maximum coolant flow rate, as described by eq 4.4.4. In order to evaluate the effectiveness of these countermeasures, we conducted tests with response times of 10 min and 20 min. These time intervals correspond approximately to half of the residence time and one full residence time, respectively.

**Figure 10:**
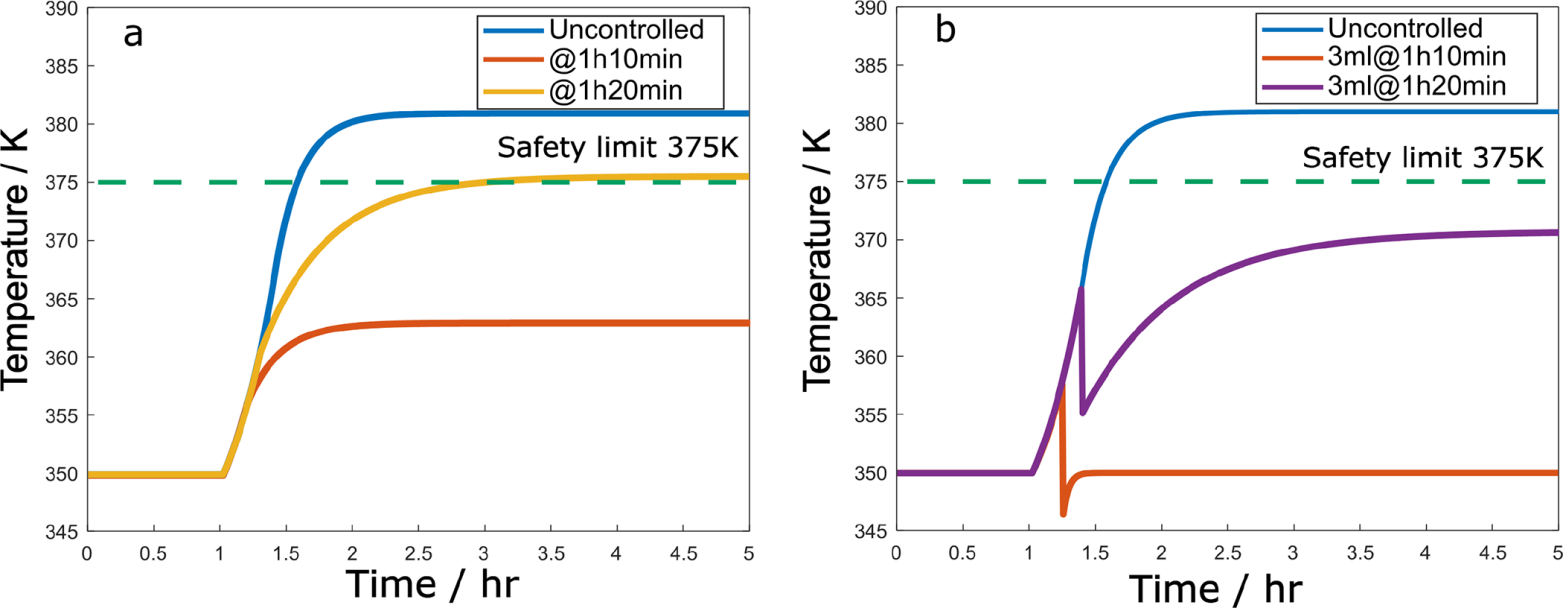
Reactor temperature vs time for a 50% step reduction in maximum coolant flow rate under (a) adjustment of feed flow rate at different times after the fault happens and (b) cold solvent injection with 3 mL solvent volume.

Figure 10a shows the response of the reactor temperature to this 50% failure, when the reactor is left uncontrolled and when a backup P controller is activated, which manipulates the feed flow rate according to *F* = *F*_s_ + *k*_1_(*T*_s_ – *T*), with *k*_1_ = – 0.35, *F*_s_ = 2 mL/min and saturation limits *F*_min_ = 0, *F*_max_ = 2. We see that when this controller is activated 10 min after the fault happened, the temperature remains significantly below the safety limit all the time. However, if it is activated 20 min after the fault happened, the temperature is slightly above the safety constraint.

Figure 10b shows the response when the reactor is left uncontrolled and when the cold solvent is pulsed into the system, to dilute the reactor contents. Considering the heat capacity of the added solvent, this results in a significant temperature decrease and can keep the reactor safe. A 3 mL addition of solvent at room temperature after 10 min keeps the reactor temperature below the safety limit. Even if the injection time is postponed to 20 min after the fault happened, 3 mL volume is capable of enforcing the safety constraint.

The worst-case scenario is 100% failure of the cooling system (complete shut-down of flow):
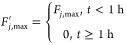
5.1.1

**Figure 11:**
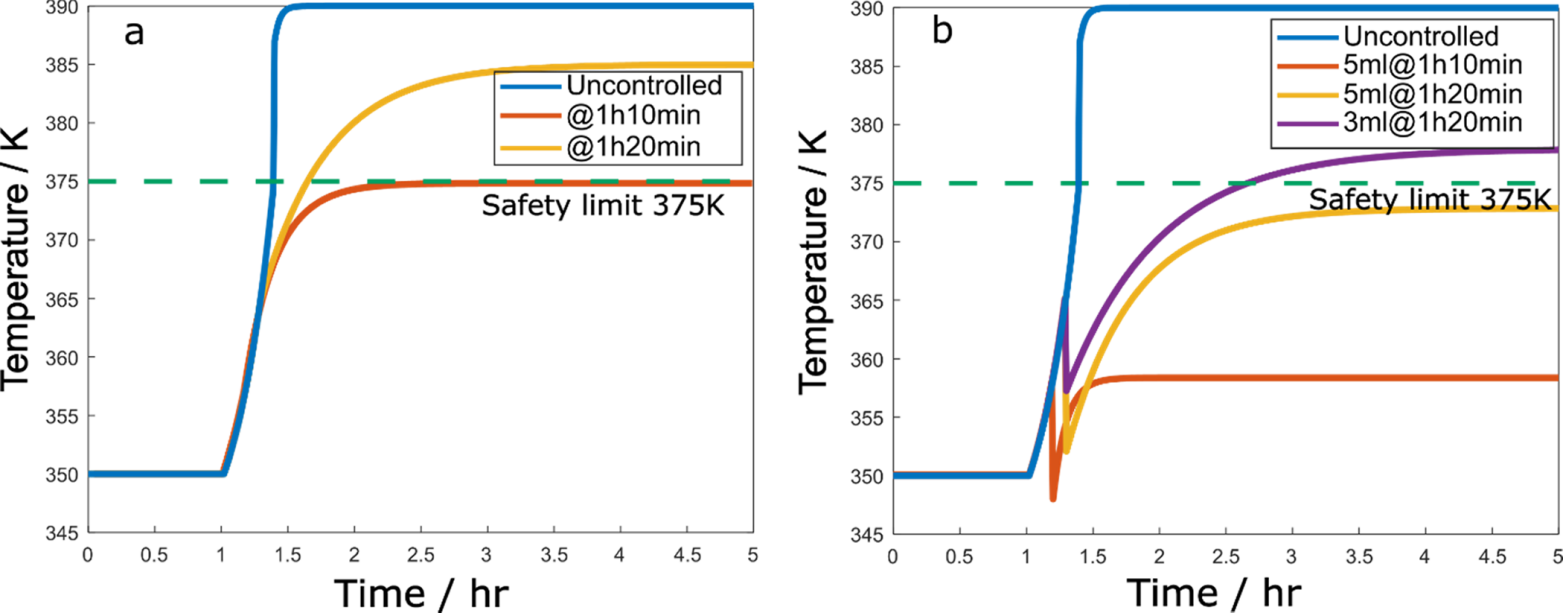
Reactor temperature vs time for a complete shut-down of the coolant flow under (a) adjustment of feed flow rate at different times after the fault happens and under (b) cold solvent injection with 3 or 5 mL solvent volume.

Figure 11a shows the response of the reactor temperature to this 100% failure, when the reactor is left uncontrolled and when the controller is switched to a P controller that manipulates the feed flow rate. In this scenario, if the controller switched 10 min after the fault happened, it could keep the reactor temperature within the safety limit all the time. However, 20 min after the fault happened is too late; the safety constraint is violated.

Figure 11b shows the response when the reactor is left uncontrolled and when the cold solvent is pulsed into the system, to dilute the reactor contents. A 5 mL addition of solvent at room temperature after 10 min or after 20 min keeps the reactor temperature below the safety limit, but if the volume is too small and/or if it happens too late (e.g., 3 mL after 20 min), the safety limit is violated.

From the simulations, it is clear that even under total shutdown of the cooling flow, it is possible to enforce the safety constraint on the temperature through the countermeasures of adjustment of feed flow rate or cold solvent injection. In the event of only partial failure (e.g., a 75 or 50% reduction of pump capacity that was considered previously), fault management is easier, allowing later intervention times or smaller injection volumes.

To summarize, the foregoing simulation results have indicated the potential effectiveness of two possible control actions, to be taken by the Fault Tolerant Control system: feed reduction and solvent injection (see Table 5). Solvent injection seems to be more effective; however, it could drive the system farther away from proper operating conditions, and thus, it would not be a good option unless it is necessary for safety. Therefore, it is natural to designate feed reduction as “Plan B”, to be activated once there is an indication of a fault, and solvent injection as “Plan C”, to be activated if there is an indication that Plan B is not effective.

**Table 5:** Fault Tolerant Control Functions to Counteract a Coolant Flow Fault

Counteraction	Manipulated input	Fault-tolerant controller parameters
feed reduction	feed flow rate	switching trigger and backup controller
solvent injection	solvent injection pulse	switching trigger and solvent volume

### 5.2 Designing Control Actions of the Fault Tolerant Controller

The simulation studies of the previous subsection show great promise, but in a real-world fault event, we surely do not have direct information on the time that the fault happened or its size. Therefore, it is not possible to pre-determine the time of activation of Plan B, or later on, of Plan C. The time of activation must be determined from real-time information, calculating appropriate online indicators, the first and foremost being the estimate of the fault size.

The estimated size of the fault will indicate the severity of the problem induced by the fault, and therefore it will be a valuable indicator to guide the course of action. However, the fault estimator takes time to converge, and if we wait until it converges, it may be too late. Therefore, the fault estimate must also be utilized *in transient*, before convergence is achieved, even though it might be under-estimating the fault size. This limitation motivates the need of using some additional online indicator that measures the level of “safeness” in transient, so that action could be prompted even when the fault estimate is still at a low level. As will be discussed in the next subsection, such an indicator could be the dynamic safety margin (DSM).

In the following two subsections, we will develop criteria for control action reconfiguration based on the two aforementioned online indicators. Simulations will be performed for different fault sizes and fault types, in order to confirm the effectiveness of the criteria in keeping the system within the safety constraints at all times.

### 5.3 DSS and DSM Analysis of the Activation Feed-Flow-Rate Adjusting Controller (Plan B)

In the event of a fault, the objective of the FTC system is to keep the process within safety constraints *at all times*. If the safety constraints are satisfied at the current time, there is no guarantee that they will be satisfied in the future, as the momentum of the system might drive it beyond the safety limits. This brings the concept of DSS, defined as the set of initial conditions that guarantee that the entire system trajectory meets the constraints, and the concept of DSM, defined as the distance from the boundary of the DSS (see Section 2.1). In the absence of a fault, the nominal controller will provide a comfortably large DSS to protect from normal process variability due to disturbances, but when a fault happens, the DSS will shrink and might even become an empty set. When a fault happens, the control system must “replace” the lost DSS (or part of DSS) due to the fault, through the appropriate countermeasures. This motivates using the DSS as a design tool for the FTC system, and the online monitoring of the DSM as a guide for deciding on the timing and the magnitude of the countermeasures, so that the system stays within DSS at all times.

In the present subsection, we will perform DSS and DSM analysis of activating the backup controller that manipulates the feed flow rate, after a cooling system fault happens. Because our system (eqs 3.1.1–3.1.4) is of fourth order, to facilitate calculations and the graphic representation of the DSS and the system trajectories, we performed two approximations. The first one is a quasi steady-state approximation on the cooling jacket, assuming that the heat transfer between the reactor and the cooling jacket is very fast, i.e., eq 3.1.4 is approximately at steady state, yielding the algebraic relation of eq 5.3.1:
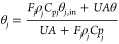
5.3.1

The second is a quasi steady-state approximation on the difference (*C*_B_ – *C*_A_) of the reactant concentrations in the reactor (excessive concentration of reactant B). This gives
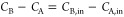
5.3.2

The resulting approximate second-order model is given by eqs 5.3.3 and 5.3.4 below:
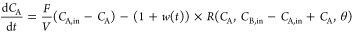
5.3.3
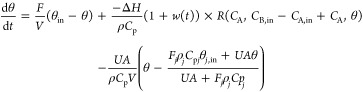
5.3.4

The model of eqs 5.3.3 and 5.3.4 was linearized and discretized, and the DSS was calculated using standard algorithms for maximal admissible sets,^44,45^ under alternative fault size scenarios and control actions. The parameters of Tables S1 and S2 were used in the simulation and the disturbance *w*(*t*) was uniformly distributed in [ – 0.05,0.05].

**Figure 12:**
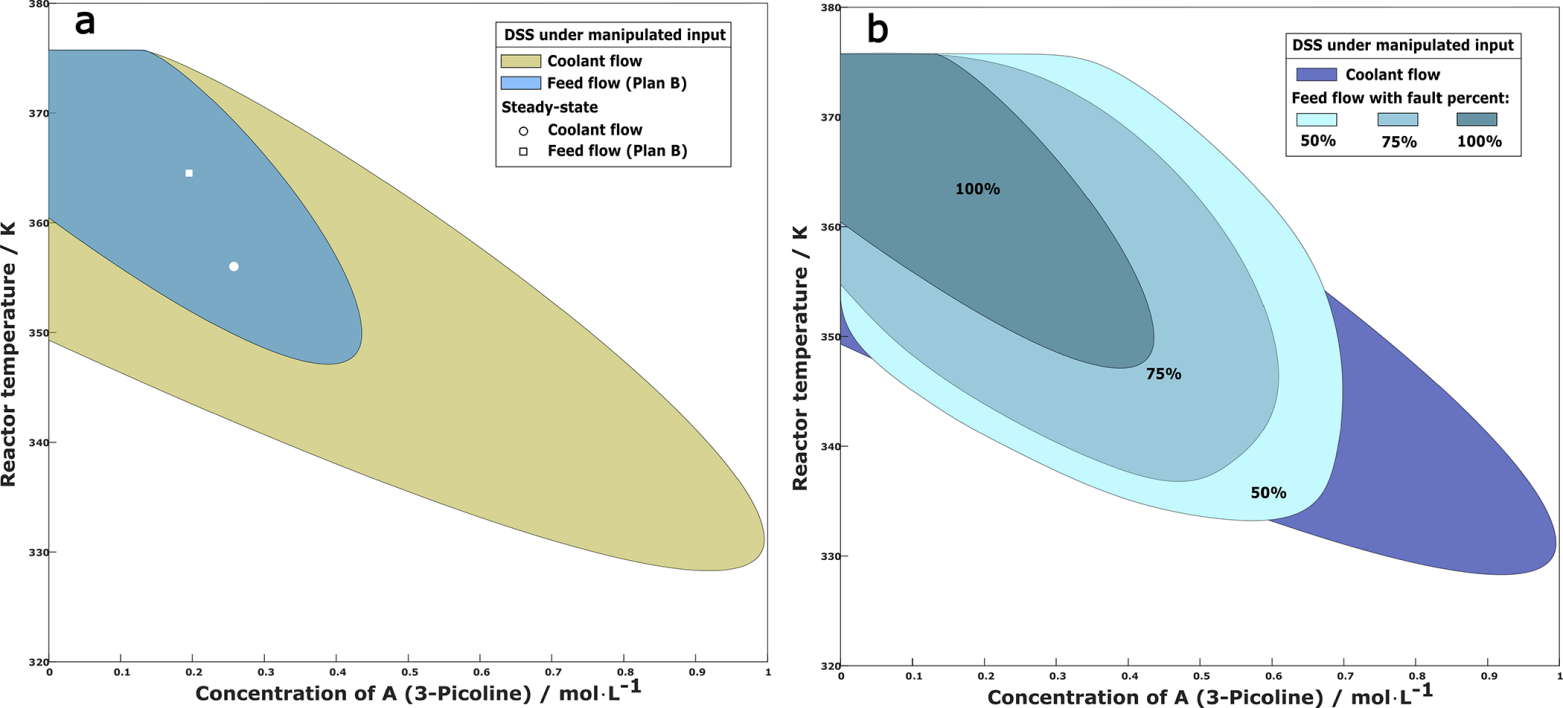
(a) DSS under the nominal PI controller in the absence of fault versus DSS under the backup P controller manipulating feed flow rate when cooling system fails (Plan B); (b) DSS under different fault sizes (different percent loss of coolant flow rate).

Figure 12a depicts the DSS under the nominal PI temperature controller that manipulates the coolant flow rate, as well as the DSS under the backup P controller that manipulates the feed flow rate in the event that the cooling system completely fails.

When there is a 100% failure in the cooling system, the DSS of the nominal controller becomes an empty set. However, we have the option to switch the controller, to manipulate the feed flow rate instead of the failed coolant flow, and this will provide a nontrivial DSS. The new DSS will be significantly smaller in size, covering a relatively small part of the nominal DSS, but still it will provide some room for controlling the process in the presence of disturbances and faults. This indicates that feed flow rate adjustment does have potential as the countermeasure to cooling system failure. When there is a partial failure of the cooling system, e.g., 50 or 75% loss of coolant flow rate, and the feed-flow-adjusting controller gets activated, there will be combined control action, leading to an overall DSS that is larger than in the case of complete failure. Figure 12b compares the combined DSS for different fault sizes (different percent loss of maximum coolant flow rate) to the nominal DSS of the fault-free system. The DSS gets larger with decreasing percent failure, but even with 50% fault, still it does not cover the entire DSS of the nominal controller. Because of this limited ability of feed flow rate manipulation, it is expected to be effective primarily for smaller-size faults.

In Figures 10a and 11a, we had seen that by activating the feed-flow-adjusting backup controller early enough, the process could be kept within the safety limit all the time. The problem is how to decide to switch, without any direct information on the time of the fault incident or the fault size. However, what we do have is a residual generator/fault estimator that can detect and estimate the fault, and the question is how to utilize its signal to decide when to switch. In this direction, a real-time calculation and monitoring of DSM is expected to be very useful, as it provides a direct indication of how far is the process from getting unsafe.

**Figure 13:**
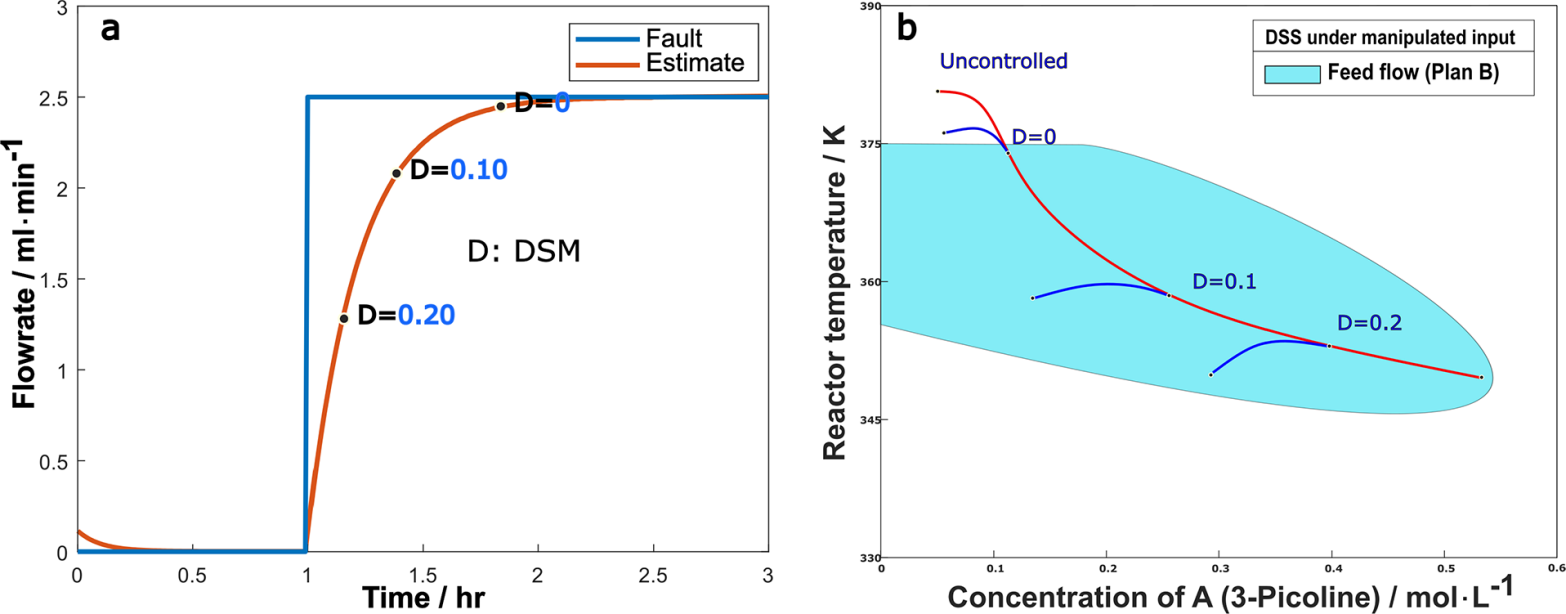
(a) Fault estimate with DSM monitoring and (b) trajectories after switching at different DSM values under 50% step reduction of maximum coolant flowrate.

Figure 13a shows the fault estimator signal under a 50% step reduction of the maximum coolant flow rate. It is the same as the one shown in Figure 7a, but with a DSM estimate marked along the time response trajectory. The DSM has been calculated assuming that only the backup feed-flow controller is active since we do not know if the cooling system underwent complete or partial failure. As time goes on, the value of the DSM decreases, and this gives a clear indication of the process moving in an unsafe direction.

Figure 13b shows the system trajectories in state space, both for the uncontrolled case (red line) where the reactor runs away, and for cases of controller switching at the time DSM assumes the value of 0.2, 0.1 or 0 (blue line branching off the red line). The system trajectories were calculated up to 4 h after the controller switching is made. Figure 13b also shows the DSS of the backup controller that manipulates the feed flow rate. We see that a DSM value of 0.2 or 0.1 is early enough for switching the controller, as the system is within the DSS of the backup controller and far enough from its boundary. However, if we wait until DSM is zero, it is too late and the system runs away, violating the safety constraint.

Additional simulations were made for smaller fault sizes (not shown here). It was found that for step faults of magnitude less than 1.8 mL/min (less than 18% loss coolant flow rate), the system trajectories satisfy the safety constraint of 375 K at all times, even without activating the backup controller. But because the fault estimator has dynamics, with the estimate lagging behind the actual fault size, it seems reasonable to take no action as long as the fault estimate is below 1.5 mL/min, a representative estimate slightly lower than 1.8.

**Figure 14:**
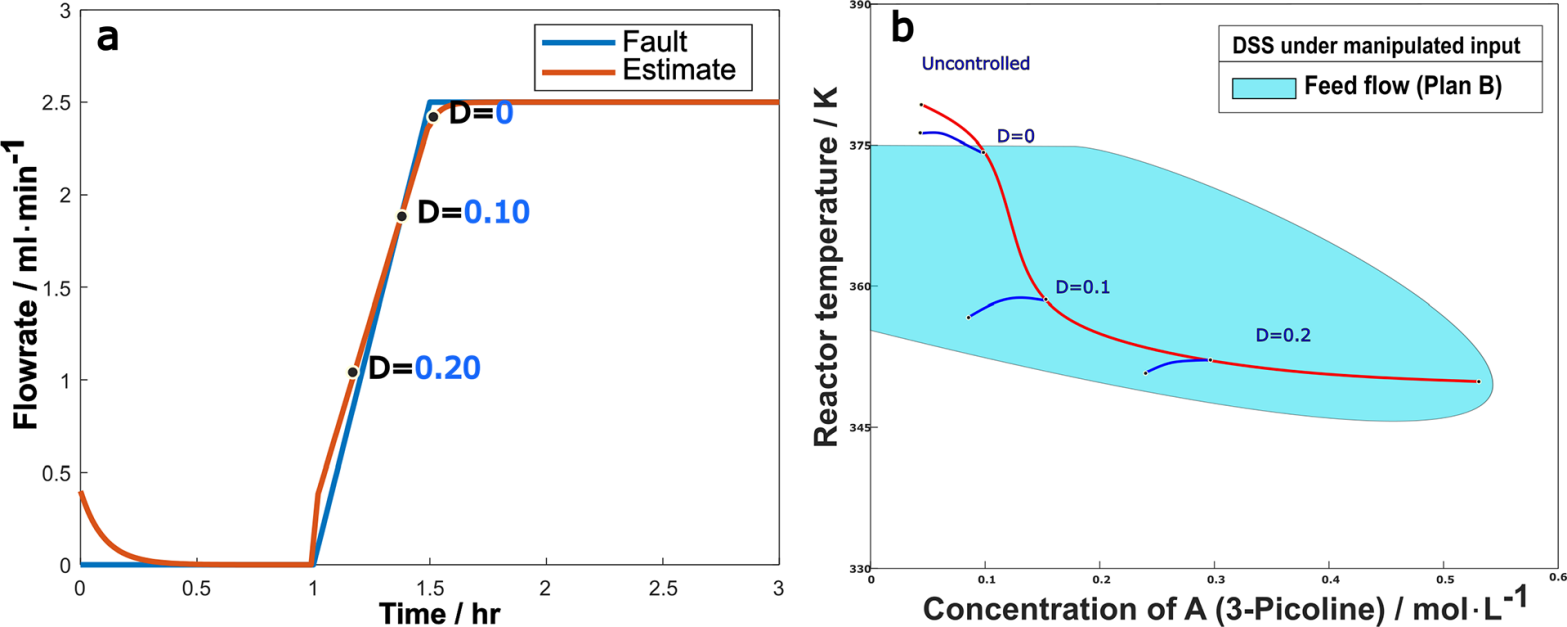
(a) Fault estimate with DSM monitoring (b) trajectories after switching at different DSM values under 50% drift reduction of maximum coolant flowrate.

The case of a drift fault, as defined in eq 4.4.6, was also examined. Figure 14a shows the fault estimator signal for a 50% drift reduction of the maximum coolant flow rate. Comparing to step faults where the estimate takes time to track an abrupt change, a drifting fault is easier to ensure precise tracking. Figure 14b shows the system trajectories in state space, both for the uncontrolled case (red line) where the reactor runs away, and for cases of controller switching at the time DSM assumes the value of 0.2, 0.1 or 0 (blue line branching off the red line). The same conclusion is reached as in the step case: a DSM of 0.2 or 0.1 is early enough to switch the controller, but a DSM of zero is too late.

The simulation results in this subsection suggest the following practical criterion for backup controller activation in FTC:

(i) Monitor the fault estimate; if it is less than 1.5, take no action.
(ii) Once the fault estimate exceeds 1.5, activate the backup feed-flow-rate controller and monitor DSM.

### 5.4 DSM and Fault Size Monitoring for Solvent Injection (Plan C)

In Section 5.1, the simulations of Figures 10b and 11b have indicated the potential of cold solvent injection to counteract a coolant flow fault, more effectively than through the adjustment of the feed flow rate. In fact, it turns out that the previously suggested strategy of switching to the feed-flow-rate controller when the fault estimate exceeds 1.5 will be sufficient as long as the actual fault size is less than 4. If the fault size is larger, this strategy will not able to keep the system within the safety limit, and solvent injection will be necessary.

To simulate the system under possible solvent injection, the dynamic equations of eq 5.3.3 and 5.3.4 will need to be modified as in eqs 5.4.1 and 5.4.2 below:
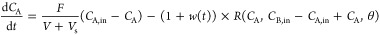
5.4.1
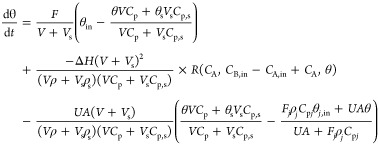
5.4.2where *V_s_* is the injection volume; it is zero before injection, and equal to the solvent volume after injection. The other parameters are as in Tables S1 and S2 and the disturbance *w*(*t*) was uniformly distributed in [ – 0.05,0.05].

**Figure 15:**
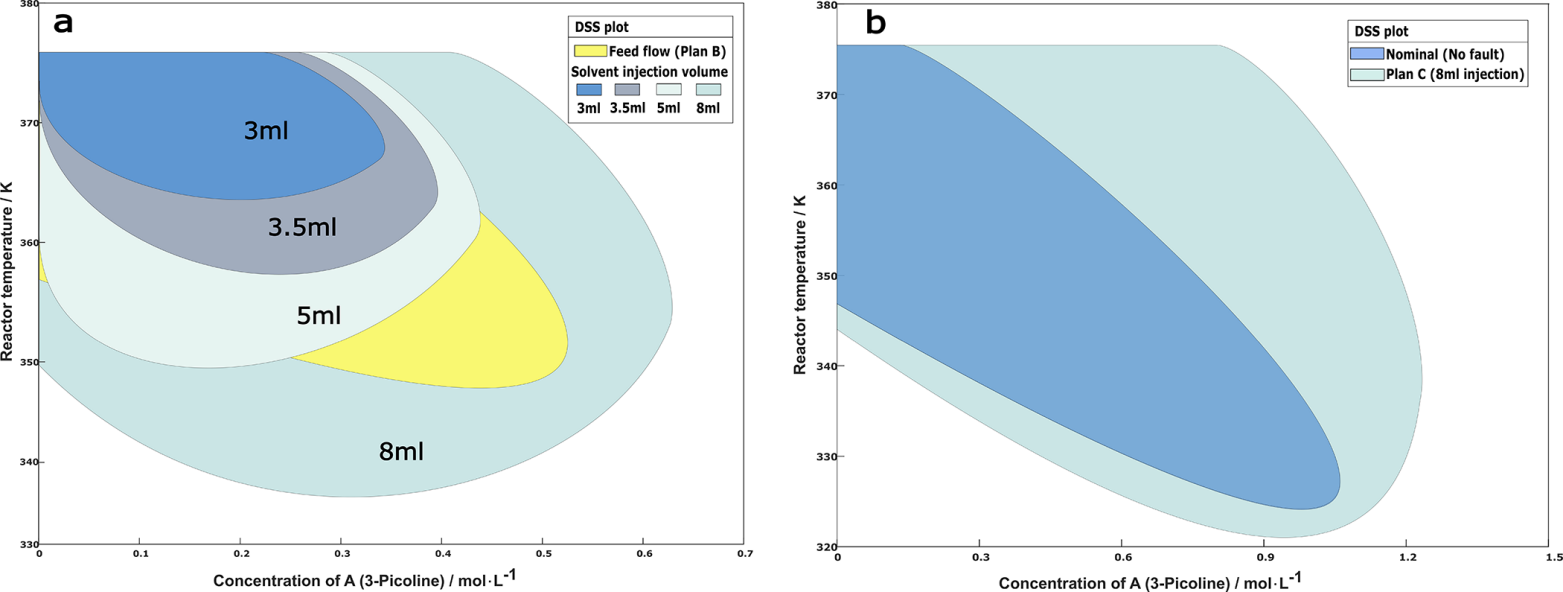
(a) DSS under different solvent injection volumes, 3, 3.5, 5 and 8 mL, compared with the DSS under adjustment of feed flow rate (b) DSS under both adjustment of feed flow rate and 8 mL solvent injection, compared with the nominal DSS.

Figure 15a depicts the DSS after solvent injection for different injection volumes of 3, 3.5, 5 and 8 mL, as well as the DSS under the P controller that manipulates the feed flow rate, all for the scenario of complete failure of the cooling line. As we can see, with the increasing amount of solvent, the DSS is also enlarged, showing a strong dependence on the quantity of infused solvent. For the specific value of 8 mL, the DSS under solvent injection is a significantly larger superset of the DSS of the backup P controller that manipulates the feed flow rate, and this indicates the stronger effect of solvent injection compared to a feed-flow rate reduction. In case the 8 mL solvent injection is combined with the feed-flow-adjusting controller, the overall DSS is much larger than the nominal DSS, as seen in Figure 15b.

**Figure 16:**
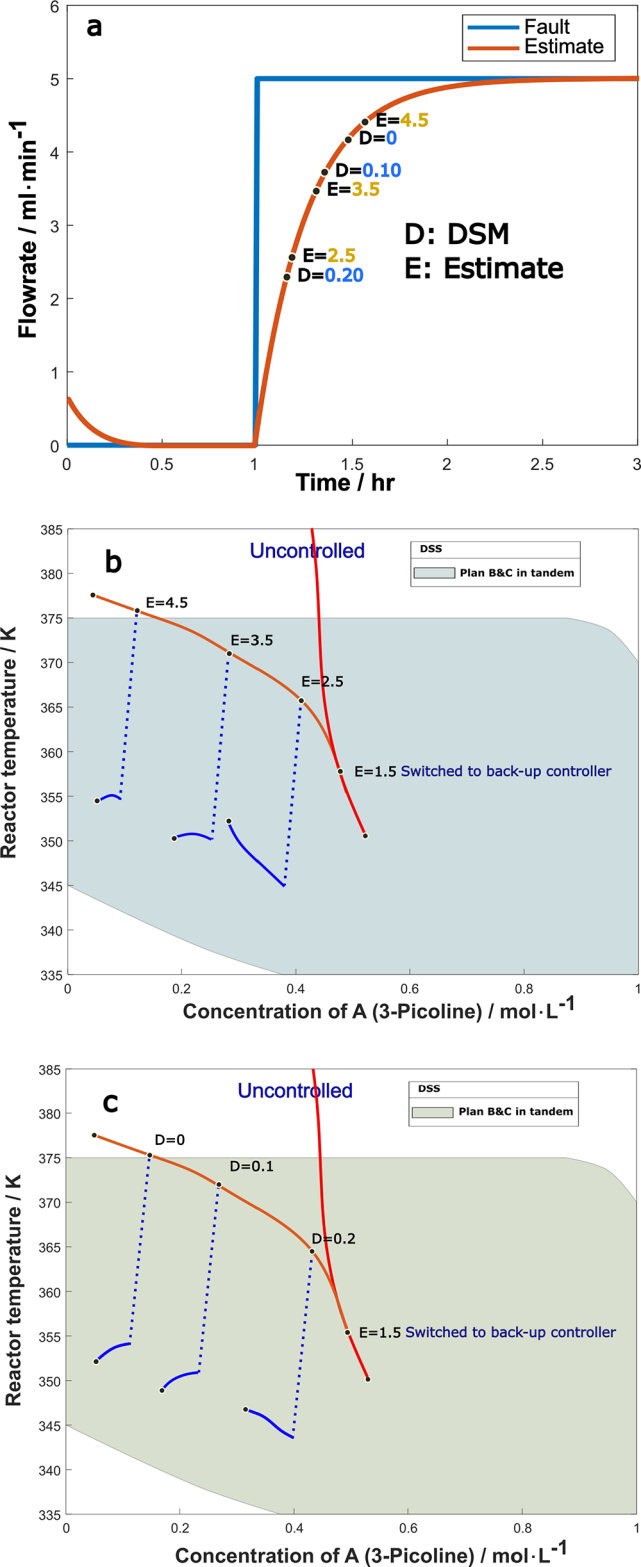
(a) Fault estimation and monitoring of DSM for 75% step reduction in coolant flow, D stands for DSM and E for fault estimate, (b) trajectories corresponding to injection at different fault estimate values, and (c) trajectories corresponding to injection at different DSM values.

Figure 16a shows the fault estimator signal under 75% step reduction of maximum coolant flow rate (eq 4.4.5), which is a major fault, with both the DSM and fault size estimate marked along the time response plot. The DSM is calculated based on the worst-case scenario, 100% coolant flow rate failure. The fault estimate approaches the actual fault size within 1 h, and during that time, the value of DSM decreases, indicating that the system is moving toward the unsafe region.

Since this is a major fault, it is important to monitor both the DSM and the fault estimate to determine the proper timing of solvent injection. As seen in the previous subsection with the activation of the feed-flow-adjusting backup controller, effectiveness depends on the precise activating moment, and the recommended strategy was to activate the backup controller once the fault estimate is over the threshold of 1.5. This strategy is effective for smaller fault sizes, but as we will see now, for larger faults, the activation of the backup controller needs to be followed by solvent injection. The results are in Figure 16b,c, where the timing of solvent injection was examined in terms of the fault estimate and DSM values marked in Figure 16a.

Figure 16b displays the system trajectories for the uncontrolled case (red line) in which the system goes to thermal runaway, the case where the feed-flow-adjusting backup controller was activated at the estimate size of 1.5 (orange line) in which the system still breaches the safety limit, as well as system trajectories for cases of solvent injection at the times of estimate readings of 2.5, 3.5 and 4.5 respectively, up to 4 h after solvent injection was deployed (blue line branching off the orange line). The figure also depicts the DSS of the combined backup controller and solvent injection, which is larger than that of the backup controller alone. It is clear that injection at the times of estimate readings of 2.5 or 3.5 is sufficiently early for the system to stay within the safety limit. However, if the injection is postponed until the estimate value of 4.5, the specific point is already over the temperature limit, regardless of the afterwards trajectory. Figure 16c shows a similar picture, except that the timing of solvent injection is examined in terms of different DSM values of 0.2, 0.1 and 0. We see that injection at a DSM value of 0.2 or 0.1 is adequate to contain the fault.

The simulation results of Figure 16 for solvent injection timing based on DSM or fault estimate monitoring have demonstrated that the backup controller alone cannot manage a large coolant flow fault, but safety could be maintained by solvent injection at the estimate value of 3.5 or DSM value of 0.1, thus these numbers may be utilized as the criteria for solvent injection timing.

**Figure 17:**
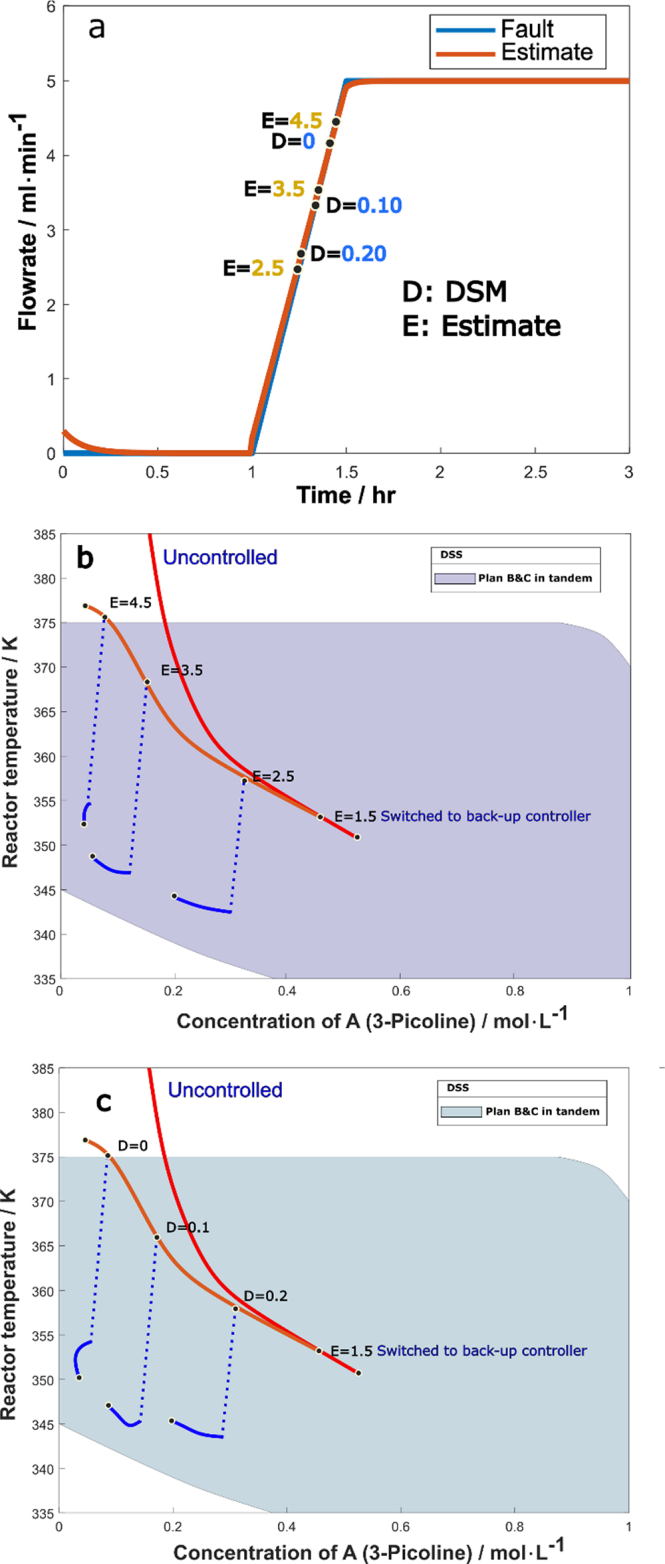
(a) Fault estimation and monitoring of DSM for coolant flow drifting down to 25% capacity, D stands for DSM and E for fault estimate, (b) trajectories corresponding to injection at different fault estimate values, and (c) trajectories corresponding to injection at different DSM.

Similarly, the case of a drift fault of eq 4.4.7 is examined. The fault estimator signal under 75% drift reduction of maximum coolant flow rate is plotted in Figure 17a, with the DSM and fault size estimate marked along the time response plot. The system trajectories in state space are traced in Figure 17b,c, where solvent injection happened when the fault estimate or the DSM assumed the values marked in Figure 17a.

Comparatively to the step fault results of Figures 16b and 17b depicts the system trajectories: the uncontrolled case (red line) which is a runaway, the case where the feed-flow-adjusting controller was activated at the estimate size of 1.5 (orange line) which is not satisfactory either, and the trajectories under solvent injection happening at fault estimate values of 2.5, 3.5 and 4.5 (blue lines branching from the orange line). We see that solvent injection at 2.5 and 3.5 keeps the system safe, but it is already too late at the value of 4.5. Likewise, the trajectories for solvent injection at DSM values of 0.2, 0.1, and 0 are shown in Figure 17c. We see that injection at a DSM value of 0.2 or 0.1 is adequate to contain the fault.

To sum up, the results in this subsection revealed the effectiveness of “plan C” for the 75% step or drift reduction of coolant flow rate in this specific system. Consequently, in the case of a larger fault, the sequence of operations would be: First, the fault estimator is implemented, and when the estimate reaches 1.5, the backup P-controller is activated to adjust the feed flow rate (as suggested in the previous subsection, “plan B”). At the same time, DSM is continuously calculated along with the fault estimate, and when the fault estimate exceeds 3.5 or the DSM value drops below 0.1, the solvent injection (“plan C”) is carried out immediately.

This plan of action is outlined in Table 6 below.

**Table 6:** Strategies for Controller Reconfiguration

Criteria for taking action	Treatment	Control action reconfiguration
estimate ≥ 1.5	reduction of feed flow rate	activation of backup P-controller adjusting feed flow rate (plan B)
DSM ≤ 0.1 or estimate ≥ 3.5	cold solvent injection	apply pulse input: injection of 8 mL of room-temperature solvent (plan C)

### 5.5 Fault Tolerant Control Actions Summarized

**Figure 18:**
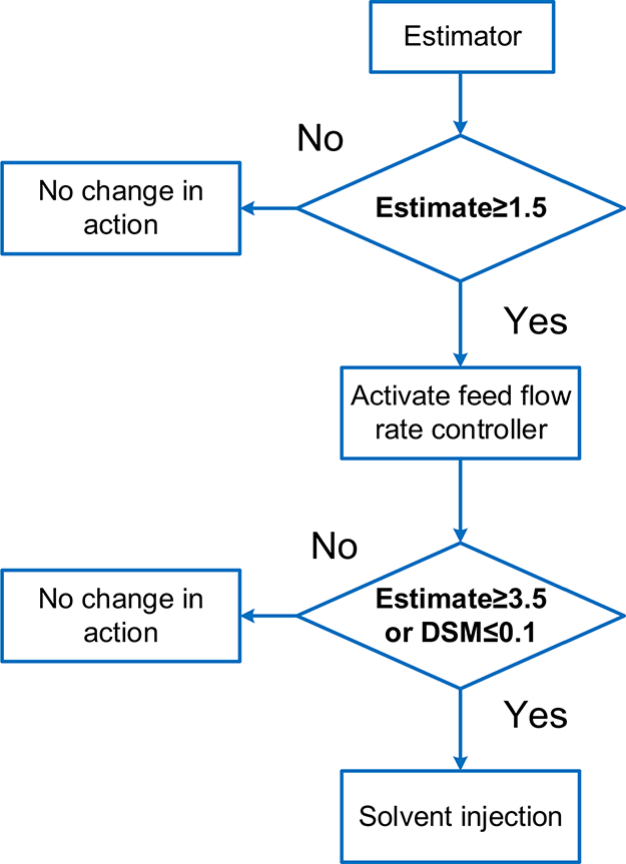
Diagram of FTC reconfiguration criteria.

The criteria in Table 6 could be represented in the diagram of Figure 18. During the process system timespan, the fault estimator keeps working to catch and estimate the size of the possible fault. At the same time, the DSM is also calculated in real-time, and both indicators are used to decide which countermeasure suits best for the specific fault that has occurred.

## 6 Conclusions and Future Work

In this work, we applied the concepts of FDI for nonlinear systems and DSS and DSM under safety constraints, and these led to the design of the FDI and controller reconfiguration components of the fault tolerant control structure. When the fault is of smaller size, the controller would switch to a simple P controller manipulating the feed flow rate, which in essence would cut down the fuel line of the exothermic reaction, and if the fault is larger, the controller would implement an emergency injection of solvent to quench the heat and reaction by dilution both in temperature and reactant concentration in addition with the feed-flow-adjusting strategy.

The proposed fault tolerant control scheme was shown to be effective in an exothermic CSTR case study. The approach is not limited to this specific reactor but has general applicability: To begin with, the dynamics and possible faults would be identified and analyzed as in Section 3; Nominal controllers and alternative fault-managing ones could be designed. Fault estimators could be built as the FDI component in the FTC, and DSS and DSM could be calculated for different faults and possible counteractions; Finally, by examining the system trajectories, a switching criteria diagram could be devised as Figure 18, leading to an overall FTC scheme.

In this study, we focused our attention on cooling flow rate faults; sensor faults will be examined in future studies, including the effect of possible interactions when the FTC strategy is trying to protect from multiple faults.

At present, we are in the process of developing an experimental setup to conduct practical tests on the proposed FTC strategy. These experiments will involve intentionally inducing faults and evaluating the performance of the FTC strategy. By carrying out this experiment, we aim at enhancing our understanding of chemical process faults in real-world scenarios. Our overarching objective is to advance the FTC strategy and ultimately integrate it into industrial applications, thereby enhancing control quality and safety in chemical industry.

## Supporting Information Available

Kinetics parameters for N-oxidation reaction; design parameters of the CSTR (PDF)
