# Supplementary material for: Model-Based Fault Diagnosis and Fault Tolerant Control for Safety-Critical Chemical Reactors: A Case Study of an Exothermic Continuous Stirred-Tank Reactor
Source: Ind Eng Chem Res. 2023 Aug 21;62(34):13554–71. doi: 10.1021/acs.iecr.3c01205 (PMC10862546; doi:10.1021/acs.iecr.3c01205)
Supplement: Supplementary file 1 — ie3c01205_si_001.pdf [file ie3c01205_si_001.pdf]

Supporting Information

Model-based fault diagnosis and fault tolerant control for safety-critical chemical reactors: A case study of an exothermic CSTR

Pu Du[1], Joshiba Ariamuthu Venkidasalapathy[2], Sunjeev Venkateswaran[1],

Benjamin Wilhite[1] and Costas Kravaris[1]*

(1) Artie McFerrin Department of Chemical Engineering, Texas A&M University,

College Station, TX 77843

(2) Viking Engineering LC, Houston, TX 77079

[*] Corresponding author E-mail: kravaris@tamu.edu

**Kinetic parameters**	A_1	exp(8.08)	L/(mol·s)
	A_2	exp(25.12)	L/(mol·s)
	A_3	exp(28.14)	L/(mol·s)
	E_1	3952	K
	E_2	7927	K
	E_3	12989	K
**Catalyst concentration**	Z	0.0021	mol/L

Table S1: The kinetics parameters for N-oxidation reaction

**Property**	**Symbol**	**Value**	**Unit**
Total feed volumetric flow rate	F	2	ml/min
3-Picoline flow rate	F_A	1.418	ml/min
Hydrogen peroxide flow rate	F_B	0.582	ml/min
Reactor liquid volume	V	50	ml
Coolant feed flow rate	F_j	7.5	ml/min
Jacket volume	Vj	17	ml
Overall heat transfer coefficient	U	36	$W/(K \cdot m^2)$
Heat transfer surface area	A	0.0075	m^2
Average density of feed	ρ	878.65	g/L
Average specific heat of feed	Cp	2.499	$J/(g \cdot K)$
Density of coolant	ρ_j	1000	g/L
Specific heat of coolant	Cp_j	4.18	$J/(g \cdot K)$
3-Picoline solution feed molar conc.	$C_{A,in}$	3.234	mol/L
H_2O_2 solution feed molar conc.	$C_{B,in}$	3.395	mol/L
Enthalpy of the reaction	ΔH	-160	kJ/mol
Reactant feed-in temperature	θ_{in}	355	K
Coolant feed-in temperature	$\theta_{j,in}$	300	K
Catalyst conc.	Z	0.002	mol/L

Table S2: Design parameters of the CSTR
